# Supplementary material for: Biological Screening and Crystallographic Studies of Hydroxy γ-Lactone Derivatives to Investigate PPARγ Phosphorylation Inhibition
Source: Biomolecules. 2023 Apr 19;13(4):694. doi: 10.3390/biom13040694 (PMC10136296; doi:10.3390/biom13040694)
Supplement: Supplementary file 1 [file biomolecules-13-00694-s001.zip › biomolecules-2274397-supplementary.pdf]

Table S1. Summary of crystallographic analysis for crystals of PPARγ-LBD in complex with **1** and **3**. Values in parentheses refer to the highest resolution shell.

	PPARγ-LBD/1	PPARγ-LBD/3
**Data collection**		
space group	*C2*	*C2*
cell dimension *a*, *b*, *c* [Å]	92.87, 59.95, 117.37	93.12, 61.32, 118.77
wavelength [Å]	0.9677	0.9677
resolution range [Å]	57.15 - 2.13	40.81 - 2.20
last shell [Å]	2.19 - 2.13	2.26 - 2.20
R_{merge} [%]	0.051 (1.317)	0.052 (1.279)
unique reflections	33410	40546
mean (*I*)/σ(*I*)	11.2 (0.6)	14.5 (0.5)
completeness	94.5 (83.2)	95.6 (85.1)
No. of molecules in asymmetric unit	2	2
**Refinement**		
resolution range [Å]	57.15 - 2.13	40.81 - 2.20
R_{work} [%]	21.6	22.4
R_{free} [%]	25.6	27.1
Bond lengths r.m.s.d. [Å]	0.007	0.012
Bond angles r.m.s.d. [deg]	0.997	1.293
**PDB ID**	8ADF	8C0C

Chemistry—Experimental data

*2-Acetamidoacetic acid (**4**).*

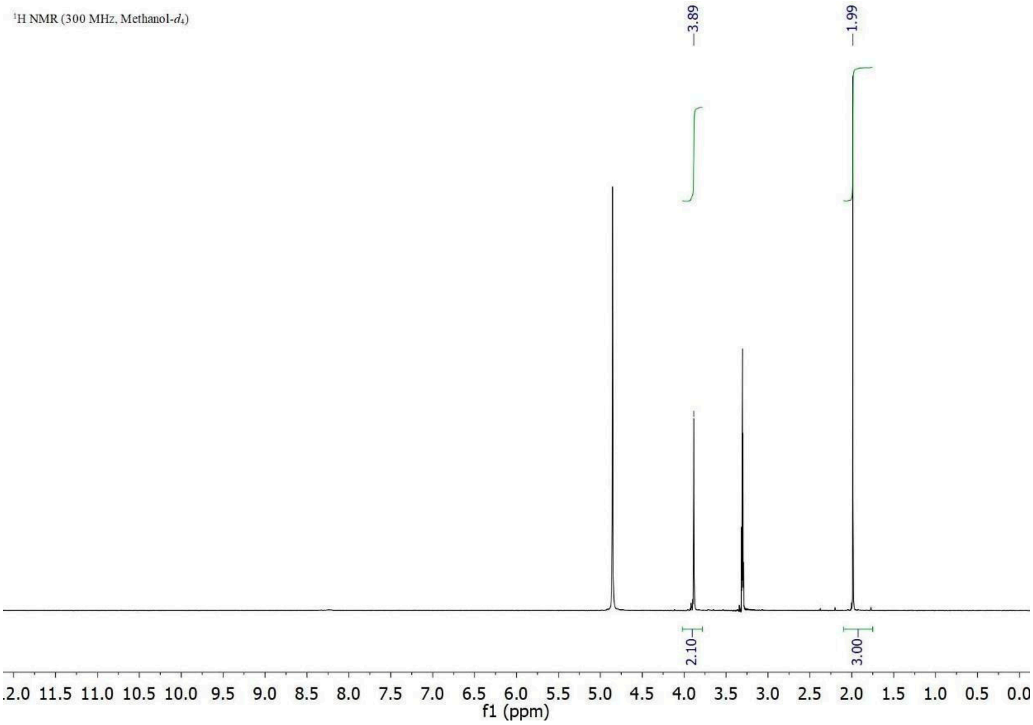

Figure S1. ^{1}H NMR spectrum of 4.

*(Z)-4-(4-Bromobenzylidene)-2-methyloxazol-5(4H)-one (**5**).*

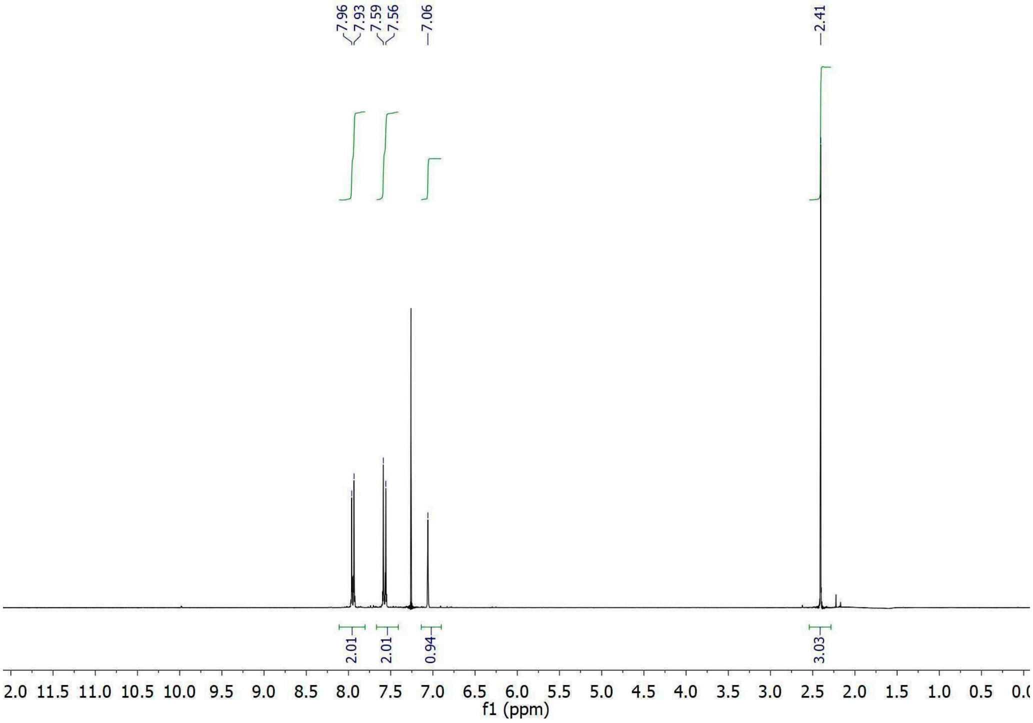

Figure S2. ^{1}H NMR spectrum of 5.

*2-Acetamido-3-(4-bromophenyl)propanoic acid (**6**).*

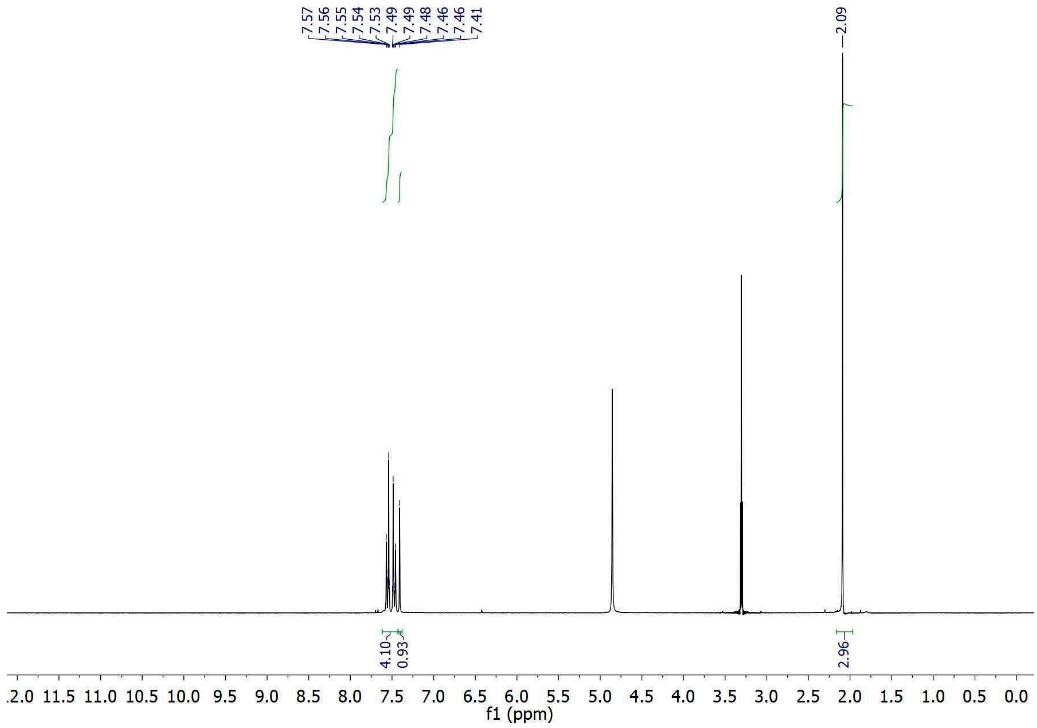

Figure S3. ^{1}H NMR spectrum of 6.

*4-(4-Bromophenyl)-3-hydroxy-5-(3-hydroxyphenyl)furan-2(5H)-one (**1**).*

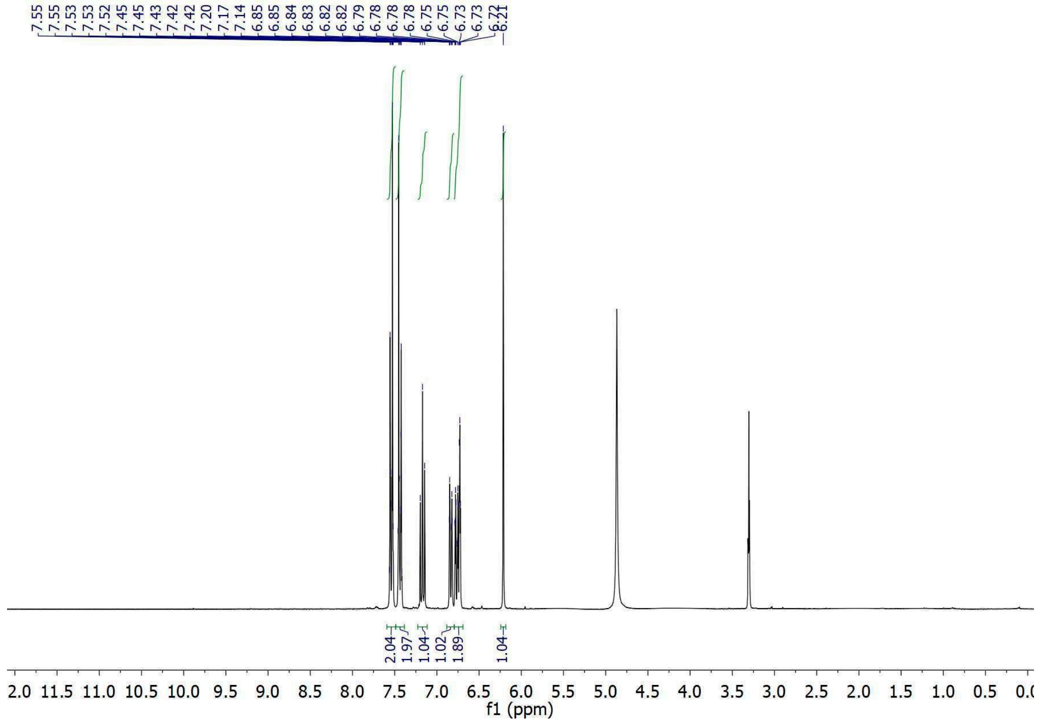

Figure S4. ^{1}H NMR spectrum of 1.

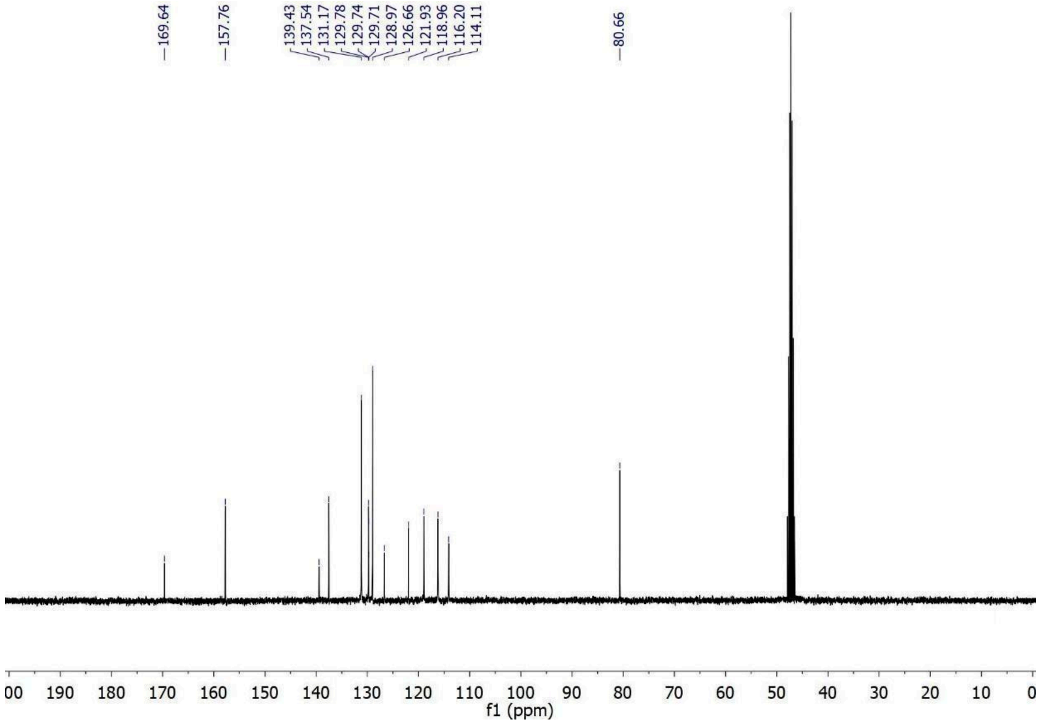

Figure S5. ^{13}C NMR spectrum of 1.

Single Mass Analysis

Tolerance = 5.0 PPM / DBE: min = -5.0, max = 300.0
Element prediction: Off
Number of isotope peaks used for i-FIT = 5

Monoisotopic Mass, Even Electron Ions
15 formula(e) evaluated with 1 results within limits (all results (up to 1000) for each mass)
Elements Used:
C: 16-16 H: 10-11 O: 4-4 Na: 0-4 K: 0-2 Br: 1-1

CZ39 2 (0.070) AM2 (Ar,40000.0,0.00,0.00); Cm (1:50) 1: TOF MS ES- 6.41e+006

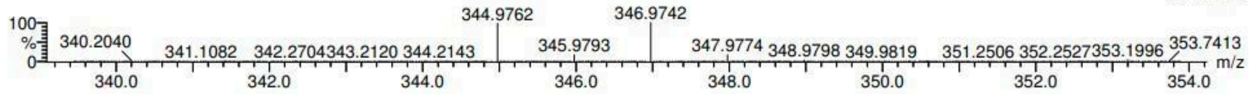

Minimum:				-5.0				
Maximum:		5.0	5.0	300.0				
Mass	Calc. Mass	mDa	PPM	DBE	i-FIT	Norm	Conf(%)	Formula
344.9762	344.9762	0.0	0.0	11.5	2820.7	n/a	n/a	C16 H10 O4 Br

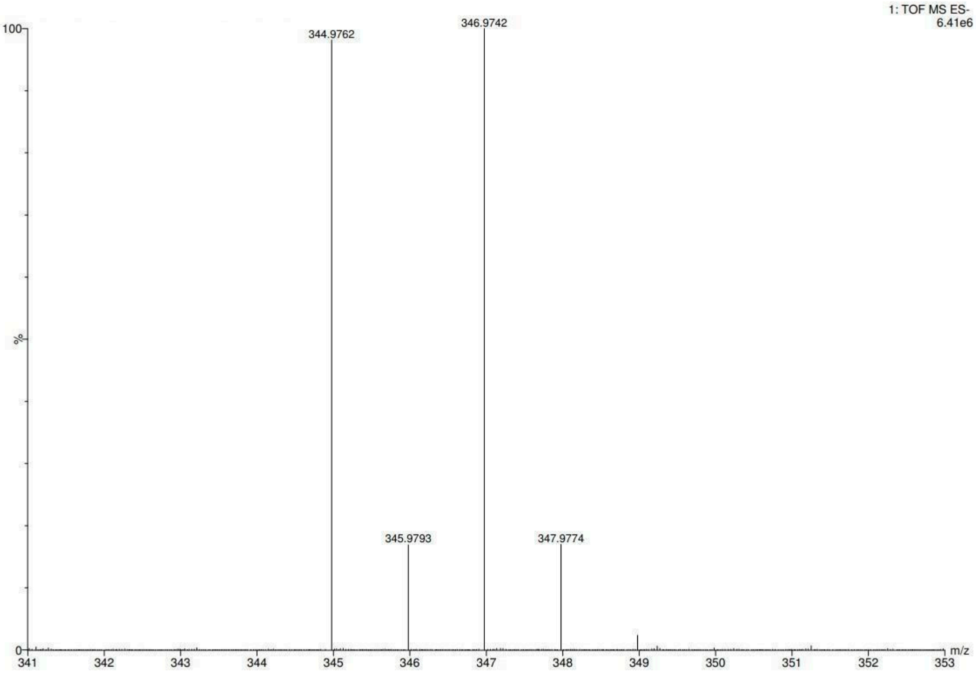

Figure S6. MS spectrum of 1.

*4-Benzoyl-3-hydroxy-5-(3-hydroxyphenyl)furan-2(5H)-one (**2**).*

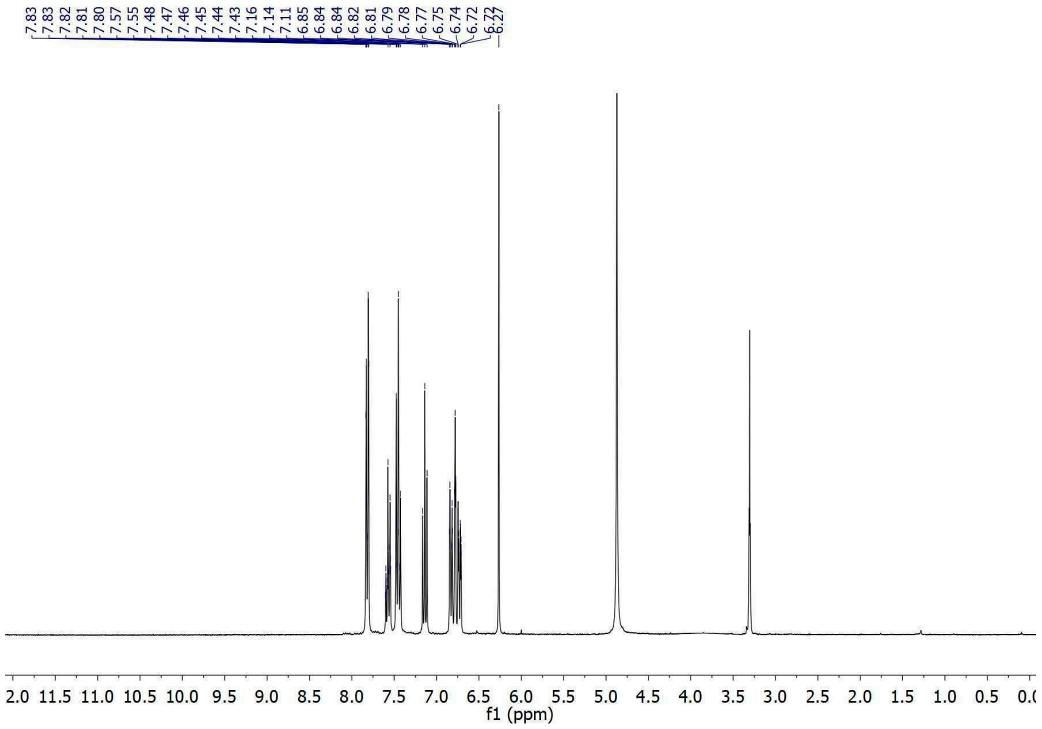

Figure S7. ^{1}H NMR spectrum of 2.

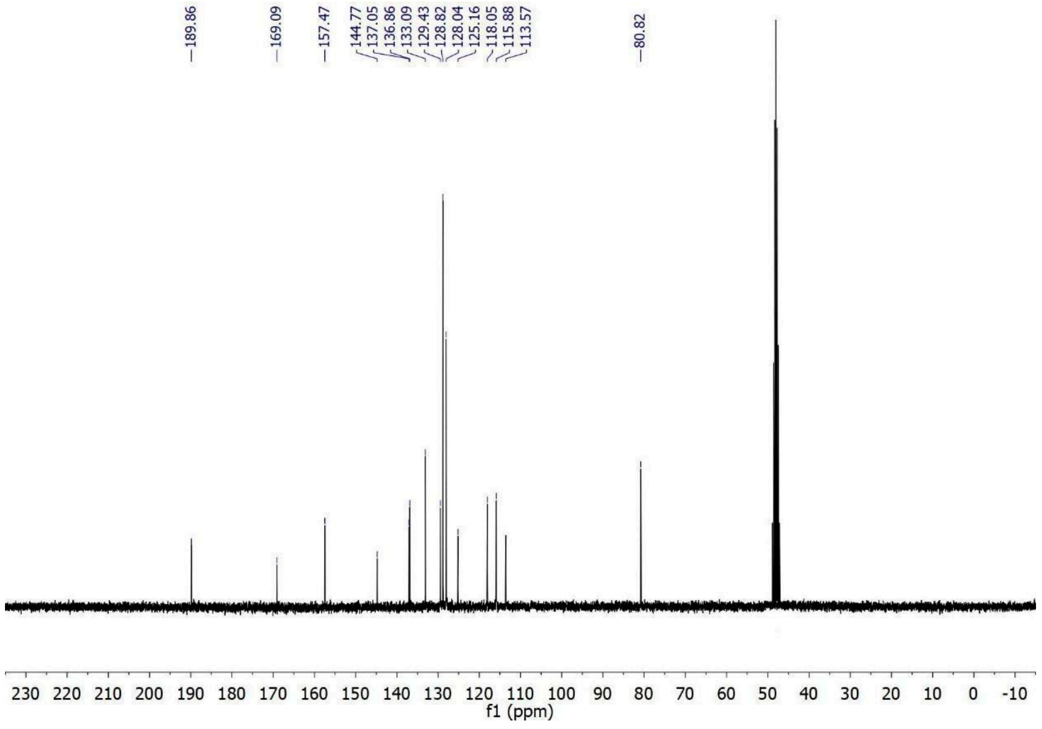

Figure S8. ^{13}C NMR spectrum of 2.

Single Mass Analysis
Tolerance = 5.0 PPM / DBE: min = -5.0, max = 300.0
Element prediction: Off
Number of isotope peaks used for i-FIT = 5

Monoisotopic Mass, Even Electron Ions
15 formula(e) evaluated with 1 results within limits (all results (up to 1000) for each mass)
Elements Used:
C: 17-17 H: 11-12 O: 5-5 Na: 0-4 K: 0-2
CZ16B 9 (0.208) AM2 (Ar,40000.0,0.00,0.00); Cm (1:50)

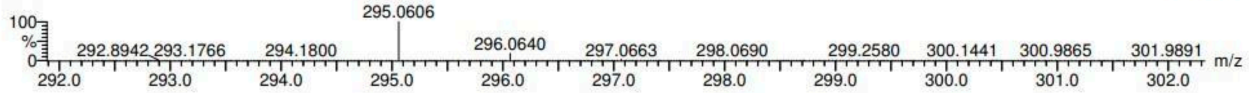

Minimum:				-5.0				
Maximum:		5.0	5.0	300.0				
Mass	Calc. Mass	mDa	PPM	DBE	i-FIT	Norm	Conf(%)	Formula
295.0606	295.0606	0.0	0.0	12.5	2960.8	n/a	n/a	C17 H11 O5

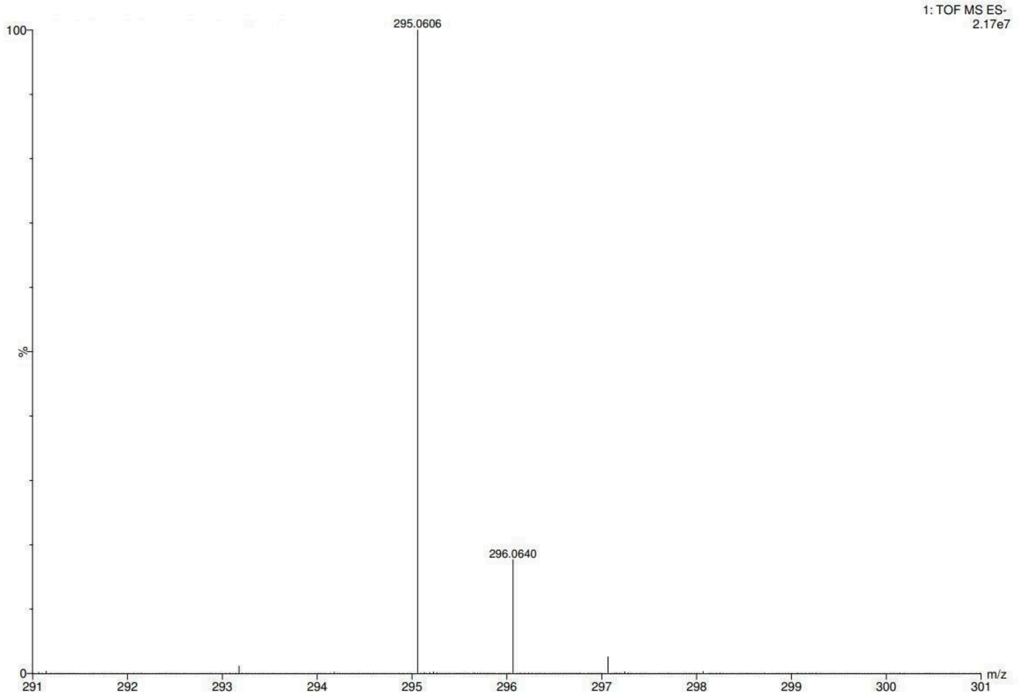

Figure S9. MS spectrum of 2.

*(Z)-methyl 2-hydroxy-3-phenylacrylate (**9**).*

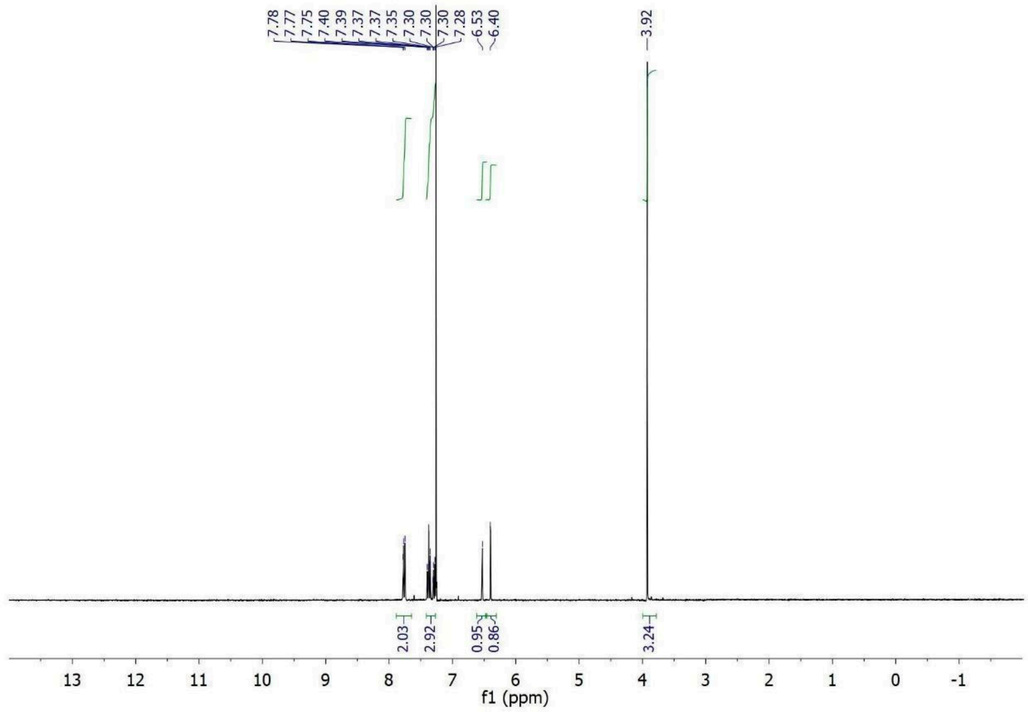

Figure S10. ^{1}H NMR spectrum of 9.

*4-(naphthalen-1-ylmethoxy)benzaldehyde (**10**).*

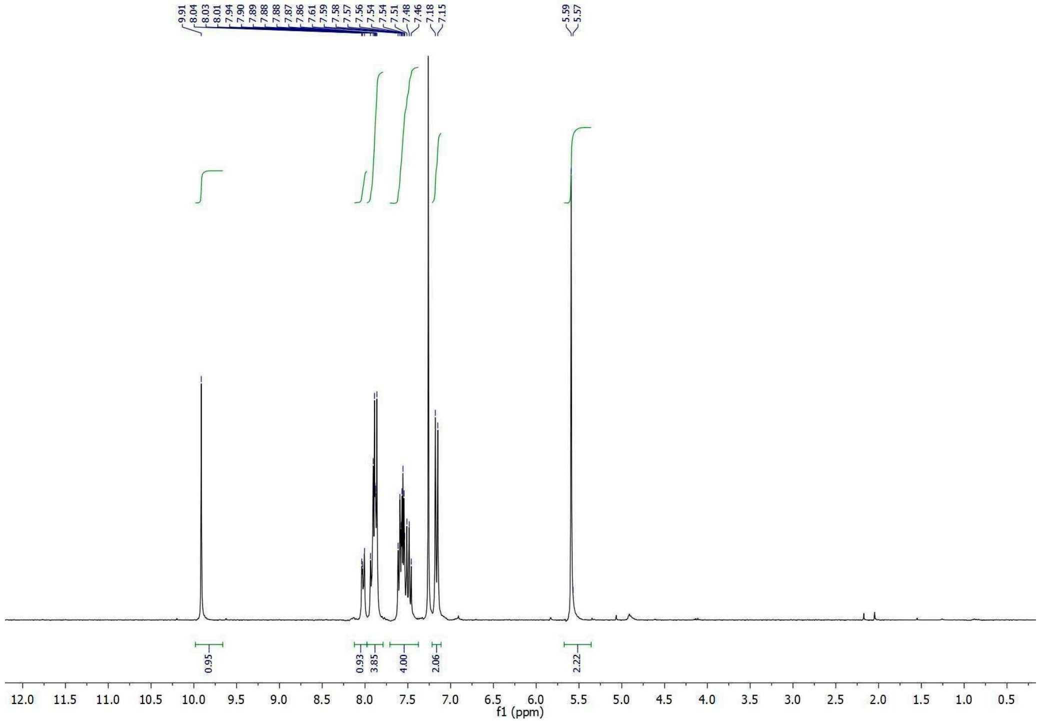

Figure S11. ^{1}H NMR spectrum of 10.

*3-hydroxy-5-(4-(naphthalen-1-ylmethoxy)phenyl)-4-phenylfuran-2(5H)-one (**3**).*

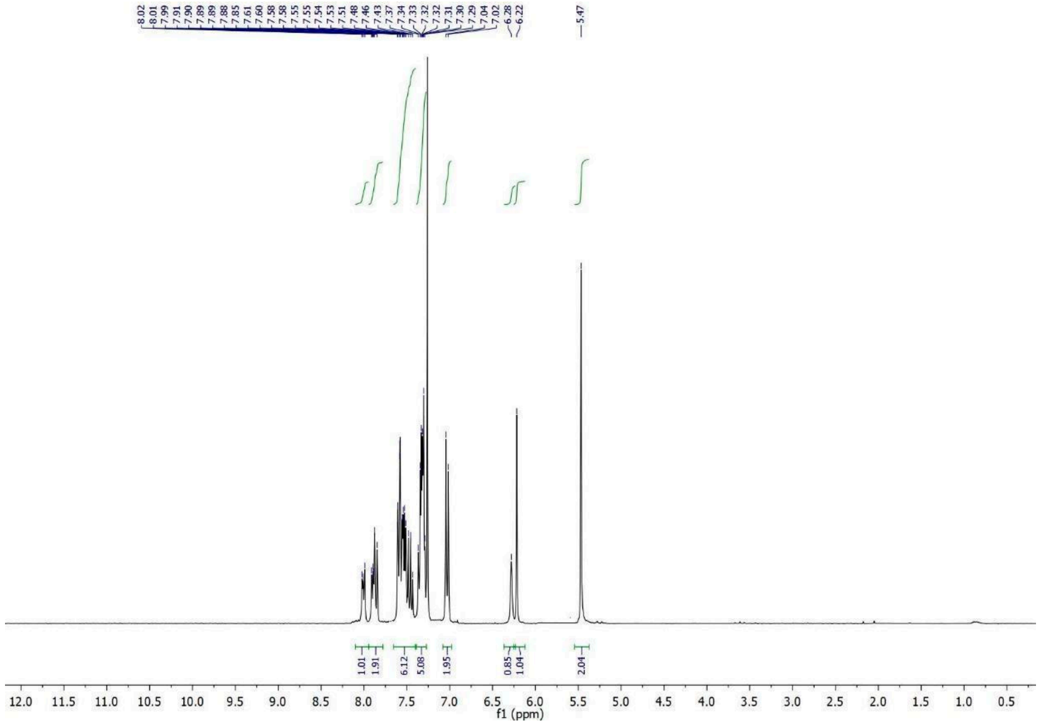

Figure S12. ^{1}H NMR spectrum of 3.

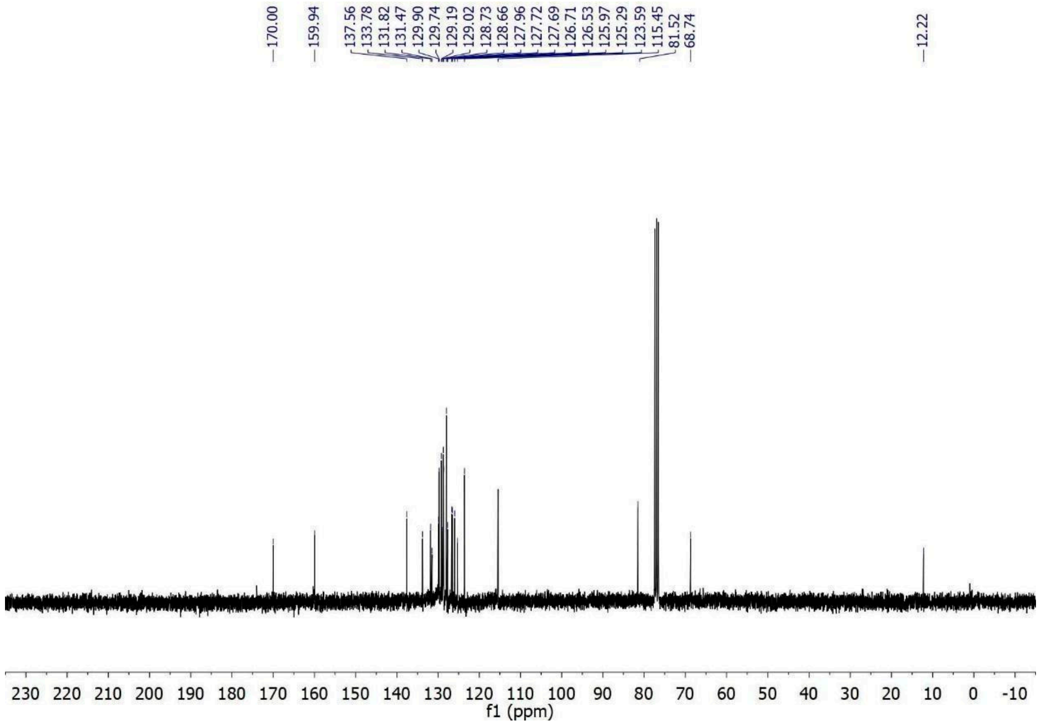

Figure S13. ^{13}C NMR spectrum of 3.

Single Mass Analysis
Tolerance = 5.0 PPM / DBE: min = -5.0, max = 300.0
Element prediction: Off
Number of isotope peaks used for i-FIT = 5

Monoisotopic Mass, Even Electron Ions
14 formula(e) evaluated with 1 results within limits (all results (up to 1000) for each mass)
Elements Used:
C: 27-27 H: 19-20 O: 4-4 Na: 0-4 K: 0-2
CZ46 2 (0.070) AM2 (Ar,40000.0,0.00,0.00); Cm (1:50)

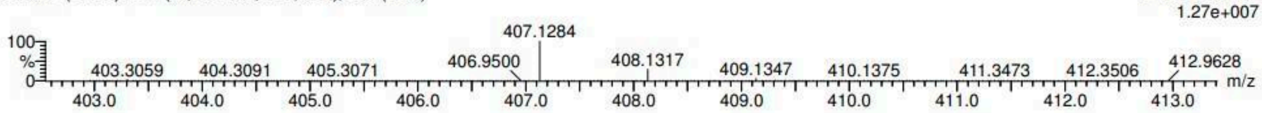

Minimum:				-5.0				
Maximum:		5.0	5.0	300.0				
Mass	Calc. Mass	mDa	PPM	DBE	i-FIT	Norm	Conf(%)	Formula
407.1284	407.1283	0.1	0.2	18.5	2291.5	n/a	n/a	C27 H19 O4

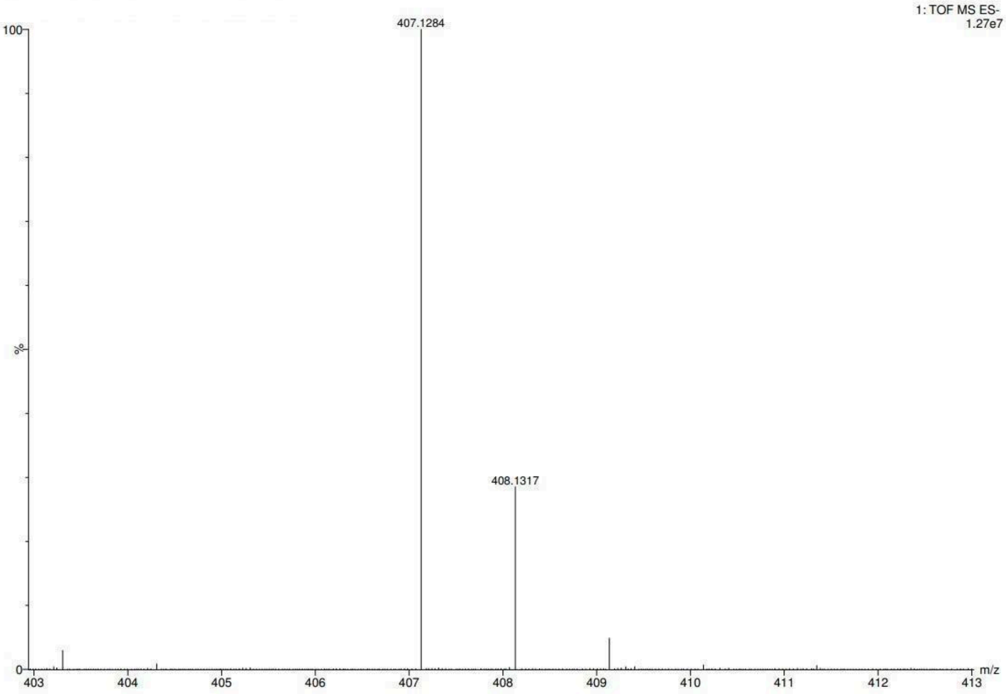

Figure S14. MS spectrum of 3.

Table S2. OneStep assay (duplicate) performed on the compounds—single gradient injection up to 10 uM Flux 50 ul/min. Dissociation 600′′. No regeneration. LT175 as the positive control.

Ligand	K_D(µM)	Structure	Sensorgram (OneStep®)
LT175 (***ref***)	2.55 ± 0.02	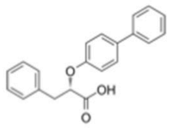	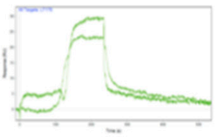
CZ39 (***1***)	2.23 ± 0.04	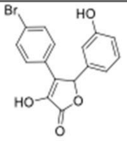	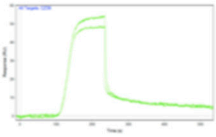
CZ16B (***2***)	1.46 ± 0.01	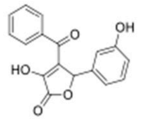	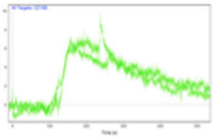
CZ46 (***3***)	3.58 ± 0.01	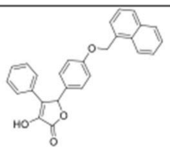	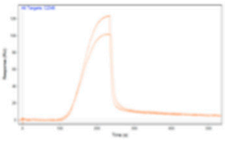
CZ30	uncertain	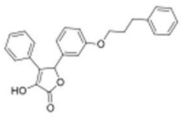	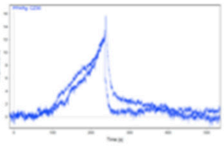
CZ41	no binding	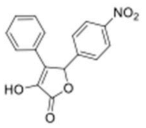	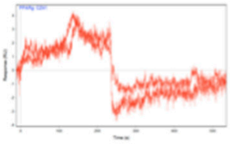
CZ43	>10 µM	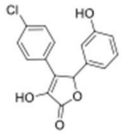	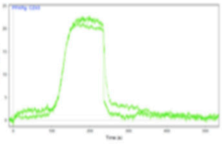
CZ48	>10 µM	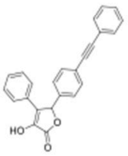	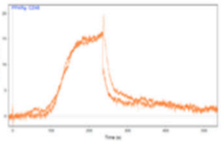
CZ49	uncertain	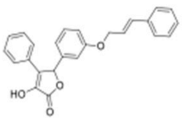	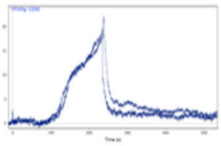

CZ52	>10 µM	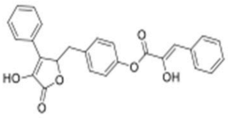	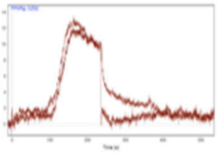
MQ1	>10 µM	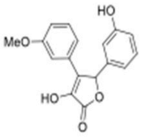	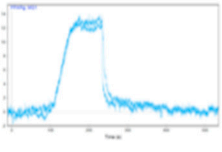
MQ5	no binding	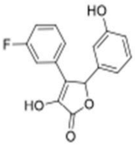	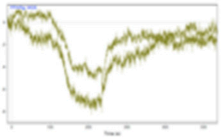
TT1	>10 µM	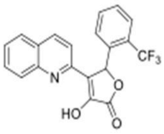	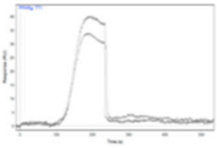
CZ11B	no binding	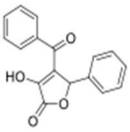	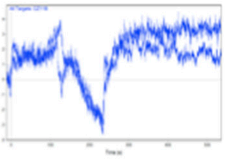
CZ13	no binding	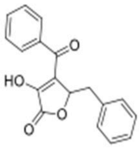	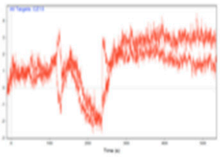
CZ18	no binding	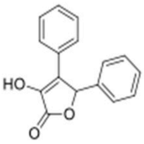	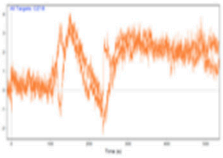
CZ19A	uncertain	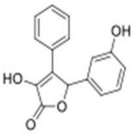	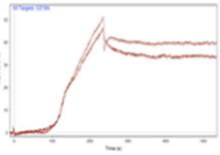
CZ19	>10 µM	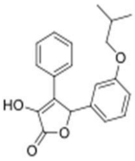	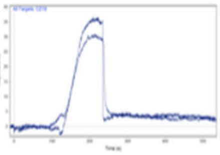

CZ21	>10 µM	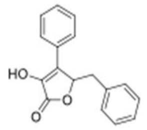	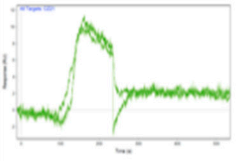
CZ22	>10 µM	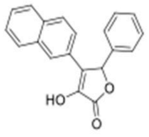	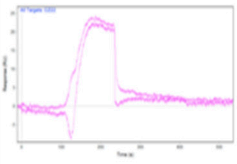
CZ27	uncertain	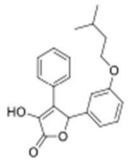	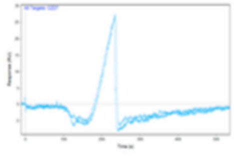
CZ28	>10 µM	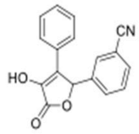	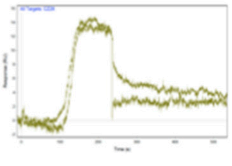
CZ29	uncertain	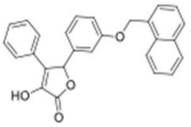	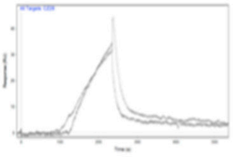
CZ31	no binding	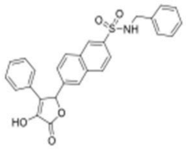	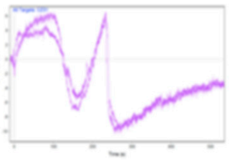
CZ32	uncertain	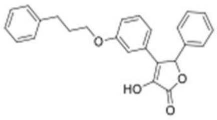	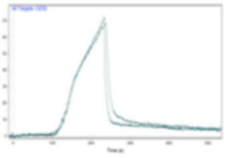
CZ35	uncertain	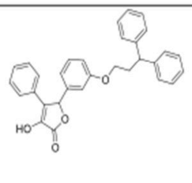	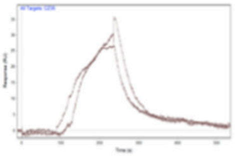
CZ36	uncertain	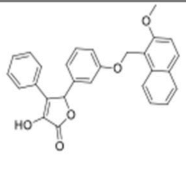	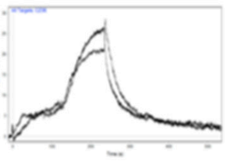

CZ37	uncertain	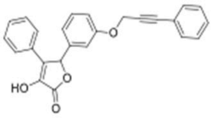	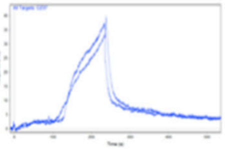
CZ38	no binding	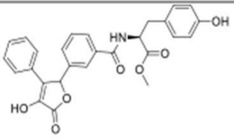	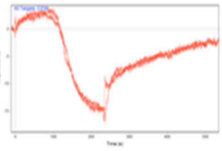
CZ47	>10 µM	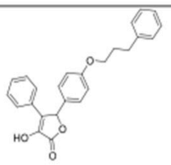	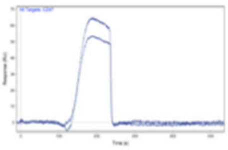
